# Analysis of the microbial content of probiotic products commercialized worldwide and survivability in conditions mimicking the human gut environment

**DOI:** 10.3389/fmicb.2023.1127321

**Published:** 2023-05-05

**Authors:** Emilia Ghelardi, Diletta Mazzantini, Francesco Celandroni, Marco Calvigioni, Adelaide Panattoni, Antonella Lupetti, Beatrice Bois De Fer, Marcos Perez

**Affiliations:** ^1^Department of Translational Research and New Technologies in Medicine and Surgery, University of Pisa, Pisa, Italy; ^2^Sanofi, Paris, France; ^3^Sanofi, Frankfurt, Germany

**Keywords:** quality control, living microbes, species identification, gastrointestinal survivability, probiotics

## Abstract

**Introduction:**

Probiotics are living microorganisms that, when administered in adequate amounts, confer a health benefit on the host. Adequate number of living microbes, the presence of specific microorganisms, and their survival in the gastrointestinal (GI) environment are important to achieve desired health benefits of probiotic products. In this *in vitro* study, 21 leading probiotic formulations commercialized worldwide were evaluated for their microbial content and survivability in simulated GI conditions.

**Methods:**

Plate-count method was used to determine the amount of living microbes contained in the products. Culture-dependent Matrix-Assisted Laser Desorption/Ionization–Time of Flight Mass Spectrometry and culture-independent metagenomic analysis through 16S and 18S rDNA sequencing were applied in combination for species identification. To estimate the potential survivability of the microorganisms contained in the products in the harsh GI environment, an *in vitro* model composed of different simulated gastric and intestinal fluids was adopted.

**Results:**

The majority of the tested probiotic products were concordant with the labels in terms of number of viable microbes and contained probiotic species. However, one product included fewer viable microbes than those displayed on the label, one product contained two species that were not declared, and another product lacked one of the labeled probiotic strains. Survivability in simulated acidic and alkaline GI fluids was highly variable depending on the composition of the products. The microorganisms contained in four products survived in both acidic and alkaline environments. For one of these products, microorganisms also appeared to grow in the alkaline environment.

**Conclusion:**

This *in vitro* study demonstrates that most globally commercialized probiotic products are consistent with the claims described on their labels with respect to the number and species of the contained microbes. Evaluated probiotics generally performed well in survivability tests, although viability of microbes in simulated gastric and intestinal environments showed large variability. Although the results obtained in this study indicate a good quality of the tested formulations, it is important to stress that stringent quality controls of probiotic products should always be performed to provide optimal health benefits for the host.

## 1. Introduction

Probiotics are living microorganisms that, when administered in adequate amounts, confer a health benefit on the host (FAO/WHO Working Group Report, 2002). Numerous probiotic products contain lactic acid bacteria belonging to the *Lacticaseibacillus*, *Lactiplantibacillus*, *Lactobacillus*, *Bifidobacterium*, and *Streptococcus* genera (Hanchi et al., 2018; National Institutes of Health, 2022). Spore-forming bacteria of the genus *Bacillus* are also commonly used as they are resistant to the harsh gastrointestinal (GI) conditions (Elshaghabee et al., 2017; Jeżewska-Frąckowiak et al., 2018). Among yeast, *Saccharomyces boulardii* exhibits a variety of beneficial properties and is been used as a probiotic microbe since several decades (Sen and Mansell, 2020).

Studies have highlighted the ability of probiotics to improve the gut-barrier function, modulate gut microbiota, enhance host immune response, exert antimicrobial activities, and possibly reduce risk, duration, or severity of diseases (Bron et al., 2017; Hu et al., 2017). Few probiotic strains have been shown to exert positive effects on obesity, diabetes, cancer, and allergies, to lower blood cholesterol levels, and to contribute to the maintenance of urogenital, oral, and central nervous system health (Manzoor et al., 2022). Further, probiotics are increasingly used to improve metabolic health, and their role has also been studied in several GI disorders (Embleton et al., 2016; Kim et al., 2019; Sanders et al., 2019; Stavropoulou and Bezirtzoglou, 2020), particularly in infections, microbiome dysbiosis, and gut-barrier perturbation.

The sum of microbial quality and functional properties of a probiotic product determines its effectiveness (Huys et al., 2013). While functional properties may depend on body site, mode of delivery and population, microbial quality assessment is more straightforward (Huys et al., 2013). To achieve an expected optimal health benefit of probiotics, it is important to ensure that adequate number and certain microbial species and strains are present in the formulations and that a sufficient amount of these microbes remain viable in the GI environment (Mazzantini et al., 2021). As such, it is imperative to assess whether the microbial composition of the probiotic is consistent with the labeled information, as different microbes may show variable health effects (van den Akker et al., 2020; Mazzantini et al., 2021; Manzoor et al., 2022). In addition, this would help in the identification of potential pathogenic contaminants that may constitute a risk to the consumer.

Quality control of probiotics is encouraged by several organizations worldwide [FAO/WHO Working Group Report, 2002; Kolaček et al., 2017; International Scientific Association for Probiotics (ISAPP), 2022]. The European Society for Pediatric Gastroenterology, Hepatology and Nutrition (ESPGHAN) recently emphasized on stringent and systematic quality control of commercial probiotic products to confirm the viability and identification of the contained microbes (Kolaček et al., 2017). Similarly, Food and Agriculture Organization (FAO) and World Health Organization (WHO) recommended that genus, species, strain designation, and minimum viable numbers of each probiotic strain at the end of the shelf-life should be marked on the product label (1).

Several techniques are available for the identification of microbes during quality control of probiotics. At this purpose, the FAO/WHO guidelines recommended the use of molecular techniques, such as DNA hybridization or 16S rRNA gene sequencing (FAO/WHO Working Group Report, 2002). Earlier studies have assessed the composition of probiotic brands marketed in specific countries and reported inconsistency between the product microbial content and the labeled information (Mazzantini et al., 2021). Further, a comprehensive study analyzing 213 microbial cultures for production of probiotic formulations reported that more than 28% were incorrectly identified because of the application of unsuitable identification methods (Huys et al., 2006). Therefore, selection of up-to-date methods that are sensitive for microbial detection and identification is essential for a robust quality control of probiotic products.

In this *in vitro* study, we evaluated the quality and survivability in the gastric and intestinal environments of microorganisms contained in 21 probiotic products commercialized worldwide.

## 2. Methods

**Figure 1:**
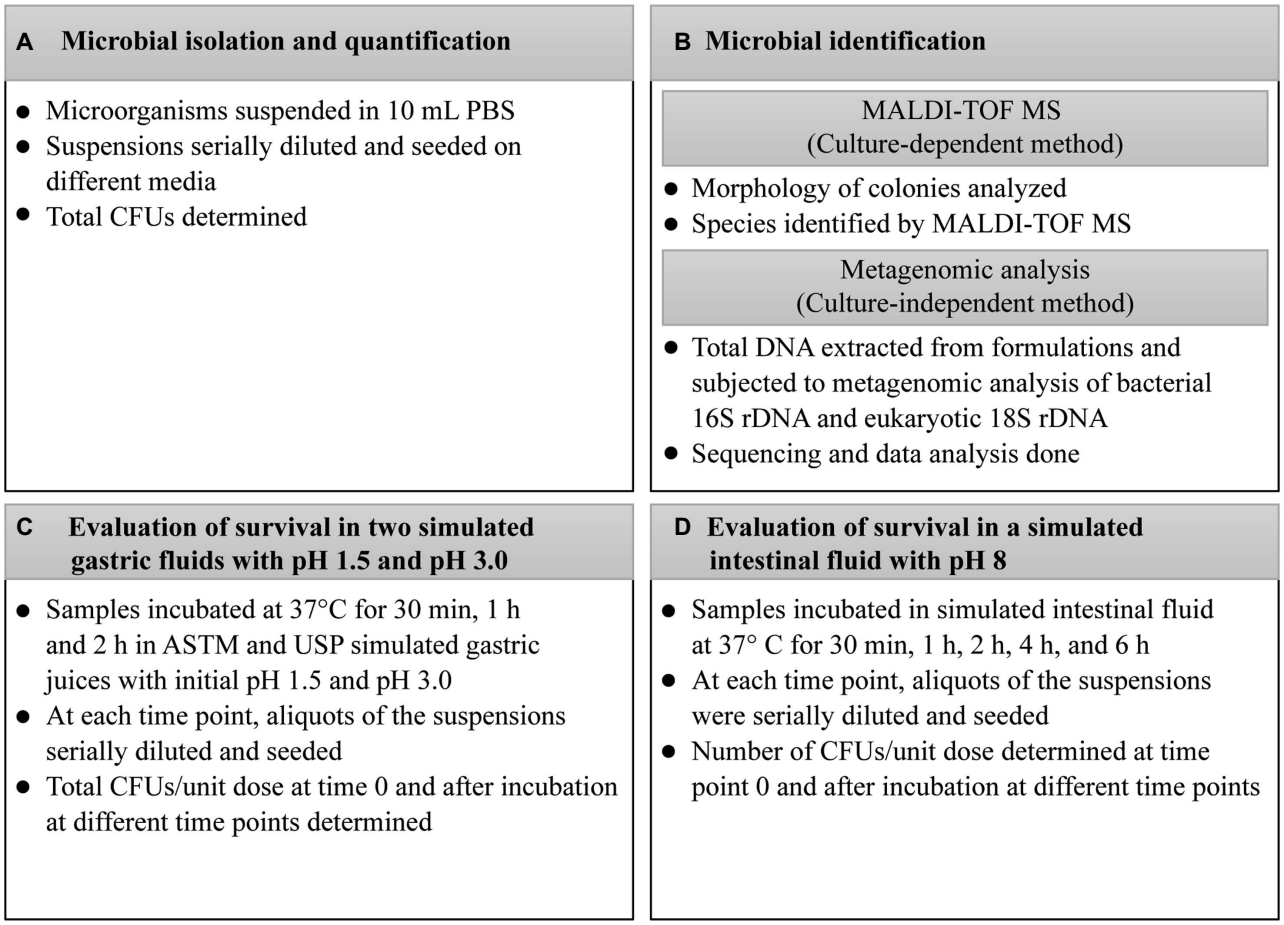
Methodology for the quantification and identification of microbes contained in probiotic products and their survival in simulated gastric and intestinal fluids. **(A)** Microbial isolation and quantification; **(B)** Microbial identification; **(C)** Evaluation of survival in two simulated gastric fluids with pH 1.5 and pH 3.0; **(D)** Evaluation of survival in simulated intestinal fluid with pH 8. ASTM, American Society for Testing and Materials; CFU, colony-forming unit; DNA, deoxyribonucleic acid; h, hour; MALDI-TOF MS, Matrix-Assisted Laser Desorption/Ionization–Time-Of-Flight Mass Spectrometry; NaCl, sodium chloride; rDNA, ribosomal deoxyribonucleic acid; USP, United States Pharmacopeia.

The probiotic products investigated in this *in vitro* study are listed in Table 1. AP, BF, CU, FD, L, LP, LR, PH, R, SD, and UL were capsules, BG, E6, ES, O, V, and Y were powders (lyophilized or orodispersible granules), CO and E4 were liquid suspensions (drops and vials, respectively), while NB was a tablet. All the products were purchased from pharmacies and analyzed within their expiration dates. Figure 1 shows an overview of the methodologies used in this study to quantify and identify microbes contained in the products, as well as to evaluate their survival in simulated acidic and alkaline fluids.

**Table 1:** Commercial probiotic products analyzed in this study.

Product	Acronym	Country of origin	Form	Expiration date
Align Probiotic Supplement Digestive Support	AP	USA	Capsule	05/2024
Bioflorin	BF	Switzerland	Capsule	05/2022
Biotics G	BG	Switzerland	Lyophilized powder	03/2022
Colidis	CO	Brazil	Drops	08/2022
Culturelle Digestive Health Daily Probiotic	CU	USA	Capsule	05/2023
Enterogermina 4B	E4	Italy	Vial	08/2022
Enterogermina 6B	E6	Italy	Orodispersible granules	10/2022
Enterolactis Plus	EP	Italy	Capsule	08/2022
Enterogermina Sporattiva	ES	Italy	Orodispersible granules	01/2023
FlorMidabìl Daily	FD	Italy	Capsule	01/2022
Linex	L	Russia	Capsule	09/2022
Lactoflorene Plus	LP	Italy	Capsule	01/2023
Lactibiane Reference	LR	Switzerland	Capsule	05/2022
Nature’s Bounty Acidophilus Probiotic	NB	USA	Tablet	09/2022
Omnibiotic 10 AAD	O	Germany	Lyophilized powder	06/2022
Phillips’ Daily Care Colon Health Daily Probiotic	PH	USA	Capsule	12/2023
RenewLife Ultimate Flora Women’s Care Probiotic	R	USA	Capsule	09/2022
Schiff Digestive Advantage	SD	USA	Capsule	03/2023
Ultra-Levure 50	UL	France	Capsule	03/2024
VSL#3	V	Italy	Lyophilized powder	10/2022
Yovis	Y	Italy	Lyophilized powder	11/2021

### 2.1. Quantification and identification of the microorganisms contained in the marketed probiotic products

#### 2.1.1. Microbial isolation and quantification

Contents of the products were suspended in 10 mL of sterile phosphate-buffered saline (PBS; 1 M KH_2_PO_4_, 1 M K_2_HPO_4_, 5 M NaCl, pH 7.2); see Figure 1A. Since E4 was an aqueous suspension of spores, vials were centrifuged at 3,800 × *g* for 10 min, supernatants were discarded, and pellets were suspended in 10 mL of PBS. Similarly, five drops of CO, corresponding to a unit dose, were added to 10 mL of sterile water, and cells were harvested by centrifugation as described above. For each product, the suspensions were seeded on Tryptic Soy Agar (TSA; Thermo Fisher Scientific, Waltham, USA) to allow the growth of all microbes contained in the formulations. For selective isolation of *Bifidobacterium* species, aliquots of the suspensions were seeded on Bifidus Selective Medium (BSM; Sigma-Aldrich, Saint Louis, USA) containing BSM supplement (Sigma-Aldrich). For selective isolation of *Lactobacillus* species, aliquots were seeded on de Man, Rogosa, and Sharpe (MRS) agar (Sigma-Aldrich). As no growth was observed by seeding aliquots of AP on TSA, BSM, and MRS, chocolate enriched agar plates (VACUTEST Kima, Padua, Italy) were specifically used for this product. TSA, BSM, MRS, and chocolate enriched agar plates were incubated for up to 72 h at 37°C in an anaerobic atmosphere using AnaeroGen™ Compact (Thermo Fisher Scientific), and in a 5% carbon dioxide (CO_2_) atmosphere using CO_2_Gen™ Compact (Thermo Fisher Scientific). For selective isolation of yeasts, aliquots were seeded on Sabouraud 2% dextrose agar (Sigma-Aldrich), and plates were incubated at 30°C for 48 h.

The number of colony-forming units (CFUs) was determined and the total CFUs contained in one dose of the original product (CFU/unit dose) were calculated. Products were considered compliant for the number of living microbes when variations in the number of CFU/unit dose ranged within −1 log compared with the labeled amount.

#### 2.1.2. Microbial identification by matrix-assisted laser desorption/ionization–time of flight mass spectrometry

Morphology of colonies was visually analyzed, and all morphologically different colonies were identified by MALDI-TOF MS in a MALDI Biotyper Microflex LT mass spectrometer (Bruker Daltonik, Germany); see Figure 1B.

A colony was directly spotted on the MALDI plate, overlaid with 1 μL of saturated α-cyano-4-hydroxycinnamic acid and air-dried. The loaded plate was then placed in the instrument according to the manufacturer’s instructions. The mass spectra were acquired within 10 min. The spectra were imported into the integrated MALDI Biotyper software (version 3.0) and analyzed by standard pattern matching with a default setting. A score ≥ 2.00 indicated identification at the species level and a score from 1.70 to 1.99 indicated identification at the genus level, whereas any score under 1.70 meant no significant similarity of the obtained spectrum with any database entry.

#### 2.1.3. Microbial identification by metagenomic analysis

##### 2.1.3.1. Total DNA extraction

Contents of probiotic products were suspended in 10 mL of sterile water before the analyses were performed Figure 1B. The E4 vials were centrifuged at 3,800 × *g* for 10 min, supernatants were discarded, and pellets were suspended in 10 mL of sterile water. For each product, cells were harvested by centrifugation at 3,800 × *g* for 10 min at 4°C. Five drops of CO were added to 10 mL of sterile water, and the cells were harvested by centrifugation as described above. All microbial pellets were suspended in 5 mL of TES buffer (5 mM EDTA, 50 mM NaCl, 30 mM Tris, pH 8.0), and then 1 mL of lysozyme (10 mg/mL) and 250 μL of ribonuclease (RNase) (10 mg/mL) were added. After incubating at 37°C for 40 min, 1 mL of 8% Triton X-100 and 10 μL of proteinase K (20 mg/mL) were added to the mixtures. The solutions were incubated at 37°C for 1 h. Then, 1.5 mL of 5 M sodium chloride (NaCl) and 1.25 mL of cetyltrimethylammonium bromide/NaCl solution (10% CTAB, 0.7-M NaCl) were added, and the mixtures were incubated at 65°C for 10 min. From each mixture, 0.5 mL was transferred to a tube, and 0.5 mL of 24:1 chloroform–isoamyl alcohol solution (Merck KGaA, Darmstadt, Germany) was added. The tubes were centrifuged at 21,000 × *g* for 10 min. Aqueous phases were transferred to new tubes and mixed with 0.5 mL of 25:24:1 phenol–chloroform–isoamyl alcohol solution (Merck KGaA). After centrifugation, aqueous phases were transferred to new tubes, mixed with 0.5 mL of 24:1 chloroform–isoamyl alcohol solution, centrifuged, and mixed with 0.6 volumes of isopropanol (Merck KGaA). The tubes were centrifuged, pellets washed with 1 mL of 70% ethanol (Merck KGaA) and extracted DNA was suspended in 50 μL of sterile water.

To perform genomic DNA extraction from yeasts, an adapted protocol was applied to BG and UL (Tavanti et al., 2007). Briefly, BG and UL powders were dissolved in 10 mL of sterile water and cells were harvested by centrifuging at 3,800 × *g* for 10 min at 4°C. Cell lysis was performed by vigorously shaking for 3 min with 0.3 g of glass beads (0.45–0.52 mm in diameter; Merck KGaA) in 0.2 mL of lysis buffer and 0.2 mL of 1:1 phenol–chloroform (Merck KGaA). After shaking, 0.2 mL of TE (10 mM Tris–HCl, 1 mM EDTA, pH 8.0) was added to the lysate. The mixture was centrifuged at 3,800 × *g* for 10 min, and the aqueous phase was transferred to a new tube. Then, 10 μL of RNAse (10 mg/mL) was added and the mixture was incubated at 37°C for 1 h; 0.5 mL of 25:24:1 phenol–chloroform–isoamyl alcohol solution (Merck KGaA) was added, and the tube was centrifuged at 21,000 × *g* for 10 min. The aqueous phase was transferred to a new tube and added with 0.5 mL of 24:1 chloroform–isoamyl alcohol solution (Merck KGaA). After centrifugation, the aqueous phase was transferred to a new tube and added with 0.6 volumes of isopropanol (Merck KGaA). The tube was centrifuged, the pellet was washed with 70% ethanol (Merck KGaA) and extracted DNA was suspended in 50 μL of sterile water. For each formulation, DNA was extracted three times in separate days.

##### 2.1.3.2. Metagenomic analysis

Genomic DNAs extracted from the products were subjected to metagenomic analysis of bacterial 16S ribosomal DNA (rDNA) and eukaryotic 18S rDNA to evaluate the microbial diversity of the products and to exclude the presence of potentially pathogenic contaminant microorganisms; see Figure 1B. Sequencing and data analysis were carried out by Novogene (Beijing, China). The V3–V4 region of the 16S rRNA gene and the V4 region of the 18S rRNA gene were amplified by using the Phusion® High-Fidelity PCR Master Mix (New England BioLabs, Ipswich, USA). Primers used for amplifications are listed in Supplementary Table S1. Polymerase chain reaction (PCR) products were purified with the Qiagen Gel Extraction Kit (QIAGEN, Hilden, Germany) and libraries generated with the NEBNext^®^ Ultra™ DNA Library Prep Kit for Illumina and quantified *via* Qubit and quantitative PCR. Amplicon sequencing was carried out on the HiSeq Illumina platform (Illumina Inc., San Diego, CA, USA). Sequence analysis was carried out using the Uparse software (version 7.0.1001). Regarding the 16S rRNA coding regions, Mothur software was run against the SSU-rRNA database of the Silva database^1^ to get the annotations about all the taxonomic ranks (i.e., kingdom, phylum, class, order, family, genus, and species). RDP Classifier (version 2.2) and Silva database were used for 18S rRNA gene sequencing.

### 2.2. Evaluation of microbial survival in two simulated gastric fluids with or without pepsin at pH 1.5 and pH 3.0

For these experiments, tablets, powders, and five drops of CO were directly inoculated in the juices. Capsules were opened and their content inoculated. Vials were centrifuged at 3,800 × *g* for 10 min, supernatants were discarded, and pellets were suspended in the simulated fluids. Samples were incubated at 37°C for 2 h at 150 rpm in the presence of two simulated gastric fluids with pH 1.5 or 3.0.The first solution was 0.07 N HCl with initial pH 1.5 or 3.0 as specified by the American Society for Testing and Materials (ASTM) [American Society of Testing Materials (ASTM), 2003]. The second solution consisted of 0.03 M NaCl, 0.084 M HCl, and 0.32% (w/v) pepsin with initial pH 1.5 or 3.0, as recommended by the United States Pharmacopeia (USP, 2003). At each time point, aliquots of the suspensions were serially diluted and seeded on TSA plates (Thermo Fisher Scientific). AP suspensions were seeded on chocolate enriched agar plates (VACUTEST Kima). The number of CFUs was determined before inclusion in simulated fluids (time 0) and after incubation at different time points – (i.e., 30 min, 1 h, and 2 h), and the CFU/unit dose of each product was extrapolated; see Figure 1C.

The evaluation of probiotic survival in gastric fluids was performed for a total of 2 h that represents a mean time for a liquid to transit in the stomach during fasting conditions (Goyal et al., 2019). In addition, experiments were performed using simulated gastric juices at pH 1.5 and 3.0 as recommended by ASTM and USP [American Society of Testing Materials (ASTM), 2003; USP, 2003].

### 2.3. Evaluation of microbial survival in a simulated intestinal fluid at pH 8

For these experiments, tablets, powders, and five drops of CO were directly inoculated in the juice. Capsules were opened and their content inoculated. Vials were centrifuged at 3,800 × *g* for 10 min, supernatants were discarded, and pellets were suspended in the simulated fluid. Samples were incubated at 37°C for 6 h at 150 rpm in simulated intestinal fluid (0.1% w/v pancreatin and 0.3% w/v Oxgall bile salts, pH 8.0). At each time point, aliquots of the suspensions were serially diluted and seeded; see Figure 1D. The number of CFUs was determined before inclusion in the simulated intestinal juice (time point 0) and after incubation at different time points (i.e., 30 min, 1 h, 2 h, 4 h, and 6 h). The CFU/unit dose of each product was extrapolated.

The evaluation of probiotic survival in the intestinal fluid was performed for a total of 6 h, representing the maximum transit time for food in the small intestine in healthy conditions (Gregersen et al., 2015).

### 2.4. Statistical analyses

#### 2.4.1. Survival of the microorganisms in marketed probiotic products in two simulated gastric fluids with or without pepsin at pH 1.5 and pH 3.0

Experiments were repeated four times on separate days, and, for each replicate, plating was performed in triplicate. The log CFU/unit dose of each repetition was first derived as the mean of triplicates at each time point. A sequential approach was followed for testing a sample at various time points as follows: time 0 versus time 2 h; time 0 versus time 1 h; and time 0 versus time 30 min (Supplementary Figure S1).

One-way analysis of variance (ANOVA) was defined as: log CFU/unit dose = time points + error.

Time points of 0 min, 30 min, 1 h, and 2 h were considered as a fixed effect and the subject as a random effect (repetition). Values were expressed as mean ± standard deviation and 95% confidence interval (CI). A two-tailed value of *p* <0.05 was considered statistically significant.

#### 2.4.2. Survival of the microorganisms in the marketed probiotic products in a simulated intestinal fluid with an alkaline pH

Experiments were repeated six times on separate days, and for each replicate, plating was performed in triplicate. The log CFU/unit dose of each repetition was derived first as the mean of triplicate at each time point. The log CFU/unit dose was assessed using one-way ANOVA defined as: log CFU/unit dose = time points + error.

Time points of 0 min, 30 min, 1 h, 2 h, 4 h, and 6 h were considered as fixed effect and the subject as a random effect (repetition). The correction of Tukey was applied to handle multiple comparisons (all pairwise comparisons). Values were expressed as mean ± standard deviation for each time point, the mean difference from ± standard error of difference, and 95% CI. A two-tailed value of *p* <0.05 was considered statistically significant.

## 3. Results

### 3.1. Quantification and identification of the microorganisms contained in the marketed probiotic products

For each product, the CFU/unit dose determined in this study and the claimed number of microorganisms in one dose are reported in Table 2. The number of living microbes contained in most of the products (i.e., AP, BG, CO, CU, E4, E6, EP, ES, FD, L, LR, NB, O, PH, R, SD, V, and Y) was comparable to that specified on the product label. As concern BF, the number of viable microorganisms was found to be higher than that labeled. However, considering that the product was analyzed before its expiration date, BF was considered compliant. LP contained a lower number of microorganisms than that declared on the product label. As regards UL, information on the number of living microbes was not labeled by manufacturers.

**Table 2:** Quantification of viable microbes contained in the analyzed probiotic products.

Product	Claimed CFUs	CFU/unit dose ± SD
AP	1.0 × 10^9^	1.80 ± 0.37 × 10^9^
BF	7.5 × 10^7^	2.08 ± 1.32 × 10^9^
BG	2.5 × 10^9^	3.54 ± 1.22 × 10^9^
CO	1.0 × 10^8^	5.31 ± 0.88 × 10^8^
CU	1.0 × 10^10^	2.83 ± 0.76 × 10^10^
E4	4.0 × 10^9^	1.59 ± 0.46 × 10^9^
E6	6.0 × 10^9^	6.66 ± 0.26 × 10^9^
EP	2.4 × 10^10^	2.38 ± 0.66 × 10^10^
ES	6.0 × 10^9^	7.70 ± 0.68 × 10^9^
FD	7.0 × 10^10^	1.61 ± 1.00 × 10^10^
L	1.2 × 10^7^	3.00 ± 0.29 × 10^7^
**LP**	**2.1 × 10**^**9**^	**1.61 ± 0.27 × 10**^**7**^
LR	1.0 × 10^10^	4.38 ± 0.94 × 10^9^
NB	1.0 × 10^8^	5.18 ± 2.10 × 10^8^
O	5.0 × 10^9^	5.15 ± 0.95 × 10^9^
PH	1.8 × 10^9^	3.03 ± 0.38 × 10^9^
R	2.5 × 10^10^	2.20 ± 0.53 × 10^10^
SD	2.0 × 10^9^	1.15 ± 0.37 × 10^9^
UL	**Not declared**	3.97 ± 1.26 × 10^8^
V	4.5 × 10^11^	3.56 ± 1.95 × 10^11^
Y	3.0 × 10^11^	5.55 ± 0.78 × 10^10^

Products that did not comply with claimed CFUs are in bold font. AP, Align Probiotic Supplement Digestive Support; BF, Bioflorin; BG, Biotics G; CO, Colidis; CFU, colony-forming unit; CU, Culturelle Digestive Health Daily Probiotic; E4, Enterogermina 4B; E6, Enterogermina 6B; EP, Enterolactis Plus; ES, Enterogermina Sporattiva; FD, FlorMidabìl Daily; L, Linex; LP, Lactoflorene Plus; LR, Lactibiane Reference; NB, Nature’s Bounty Acidophilus Probiotic; O, Omnibiotic 10 AAD; PH, Phillips’ Daily Care Colon Health Daily Probiotic; R, RenewLife Ultimate Flora Women’s Care Probiotic; SD, Schiff Digestive Advantage; SD, standard deviation; UL, Ultra-Levure 50; V, VSL#3; Y, Yovis.

All identifications carried out by MALDI-TOF MS resulted in a score > 2.00. Microbial composition of most of the products was concordant with the label claims (species were correctly identified by at least one of the used methods; see Table 3). Two exceptions were the LR and O products. In fact, LR was not compliant with the label with respect to species *Bifidobacterium longum* and *Lactobacillus helveticus*, and O did not contain the species *Bifidobacterium longum* (see Table 3). No pathogenic microbes potentially constituting a risk for the consumer health were detected in the tested formulations.

**Table 3:** Identification of the microbes contained in the probiotic products.

Product	Claimed species	No. of identified microorganisms	MALDI-TOF MS	Metagenomic analysis	Concordant with label
AP	*Bifidobacterium longum* subsp. *longum* 35,624	1	*B. longum*	*B. longum*	Yes
BF	*Enterococcus faecium* SF68	1	*E. faecium*	*E. durans*	Yes
BG	*Lactobacillus rhamnosus* W140	13	*L. rhamnosus*	*Lactobacillus* spp.	Yes
	*Lactobacillus rhamnosus* WGG		*L. rhamnosus*	*Lactobacillus* spp.	Yes
	*Lactobacillus brevis* W63		*L. brevis*	*L. brevis*	Yes
	*Lactobacillus plantarum* W1		*L. plantarum*	*L. pentosus*	Yes
	*Lactobacillus paracasei* W20		*L. paracasei*	*L. casei*	Yes
	*Lactobacillus acidophilus* W22		*–*	*Lactobacillus spp.*	Yes
	*Lactobacillus casei* W56		*–*	*L. casei*	Yes
	*Lactobacillus helveticus* W74		*–*	*Lactobacillus spp.*	Yes
	*Lactococcus lactis* W58		*–*	*L. lactis*	Yes
	*Pediococcus acidilactici* W143		*P. acidilactici*	*P. acidilactici*	Yes
	*Enterococcus faecium* W54		*E. faecium*	*Enterococcus spp.*	Yes
	*Saccharomyces boulardii* W187		*S. cerevisiae*	*S. cerevisiae*	Yes
	*Bifidobacterium animalis* W53		*–*	*B. animalis*	Yes
	*Bifidobacterium lactis* W51		*–*	*B. animalis*	Yes
	*Bifidobacterium bifidum* W23		*–*	*B. bifidum*	Yes
CO	*Lactobacillus reuterii* DSM 17938	1	*L. reuteri*	*L. reuteri*	Yes
CU	*Lactobacillus rhamnosus* GG	1	*L. rhamnosus*	*Lactobacillus spp.*	Yes
E4	*Bacillus clausii* SIN, O/C, T, N/R	1	*B. clausii*	*B. clausii*	Yes
E6	*Bacillus clausii* SIN, O/C, T, N/R	1	*B. clausii*	*B. clausii*	Yes
EP	*Lactobacillus casei* DG (*L. paracasei* CNCM I-1572)	1	*L. paracasei*	*Lactobacillus spp.*	Yes
ES	*Bacillus clausii* SIN	1	*B. clausii*	*B. clausii*	Yes
FD	*Bifidobacterium lactis* BL-04	4	*–*	*B. animalis*	Yes
	*Lactobacillus acidophilus* LA-14		*L. acidophilus*	*Lactobacillus spp.*	Yes
	*Lactobacillus paracasei* SDZ-22		*L. paracasei*	*Lactobacillus spp.*	Yes
	*Lactobacillus plantarum* SDZ-11		*–*	*L. plantarum*	Yes
L	*Lactobacillus acidophilus* (*L. gasseri*)	3	*L. gasseri*	*Lactobacillus spp.*	Yes
	*Enterococcus faecium*		*E. faecium*	*E. durans*	Yes
	*Bifidobacterium infantis*		*–*	*B. longum*	Yes
LP	*Bacillus coagulans* BC513 LGM S-24828	4	*B. coagulans*	*B. coagulans*	Yes
	*Bifidobacterium animalis* subsp. *lactis* BB12^®^ (DSM 15954)		*–*	*B. animalis*	Yes
	*Lactobacillus acidophilus* LA-5^®^ (DSM 13241)		*–*	*Lactobacillus spp.*	Yes
	*Lactobacillus casei* 431^®^ (*L. paracasei* ATCC 55544)		*L. paracasei*	*Lactobacillus spp.*	Yes
LR	*Lactococcus lactis* LA103	4	*L. lactis*	*L. lactis*	Yes
	*Streptococcus thermophilus* LA 104		*–*	*S. thermophilus*	Yes
	***Bifidobacterium longum* LA 101**		*–*	*B. animalis*	**No**
	***Lactobacillus helveticus* LA 102**		*–*	*L. casei*	**No**
NB	*Lactobacillus acidophilus* La-14	1	*L. acidophilus*	*Lactobacillus spp.*	Yes
O	*Lactobacillus rhamnosus* W71	8	*L. rhamnosus*	*Lactobacillus spp.*	Yes
	*Lactobacillus paracasei* W72		*L. paracasei*	*L. casei*	Yes
	*Lactobacillus plantarum* W62		*L. plantarum*	*L. pentosus*	Yes
	*Enterococcus faecium* W54		*E. faecium*	*Enterococcus spp.*	Yes
	*Lactobacillus salivarius* W24		*–*	*L. salivarius*	Yes
	*Lactobacillus acidophilus* W55		*–*	*Lactobacillus spp.*	Yes
	*Lactobacillus acidophilus* W37		*–*	*Lactobacillus spp.*	Yes
	*Bifidobacterium bifidum* W23		*–*	*B. bifidum*	Yes
	*Bifidobacterium lactis* W18		*–*	*B. animalis*	Yes
	***Bifidobacterium longum* W51**		**–**	**–**	**No**
PH	*Lactobacillus gasseri* KS-13	3	*L. gasseri*	*Lactobacillus spp.*	Yes
	*Bifidobacterium bifidum* G9-1		*Bifidobacterium spp.*	*B. bifidum*	Yes
	*Bifidobacterium longum* MM-2		*Bifidobacterium spp.*	*B. longum*	Yes
R	*Bifidobacterium infantis* Bi-26	10	*–*	*B. longum*	Yes
	*Bifidobacterium lactis* Bl-04		*–*	*B. animalis*	Yes
	*Bifidobacterium lactis* DSM 15954		*–*	*B. animalis*	Yes
	*Lactobacillus acidophilus* La-14		*–*	*Lactobacillus spp.*	Yes
	*Lactobacillus brevis* Lbr-35		*–*	*L. brevis*	Yes
	*Lactobacillus casei* Lc-11		*L. casei*	*Lactobacillus spp.*	Yes
	*Lactobacillus paracasei* Lpc-37		*–*	*Lactobacillus spp.*	Yes
	*Lactobacillus plantarum* Lp-115		*L. plantarum*	*L. plantarum*	Yes
	*Lactobacillus reuteri* RC-14		*–*	*L. reuteri*	Yes
	*Lactobacillus rhamnosus* GG		*L. rhamnosus*	*Lactobacillus spp.*	Yes
	*Lactobacillus rhamnosus* GR-1		*L. rhamnosus*	*Lactobacillus spp.*	Yes
	*Lactococcus lactis* Ll-23		*L. lactis*	*L. lactis*	Yes
SD	BC30 *Bacillus coagulans* GBI-30, 6,086	1	*B. coagulans*	*B. coagulans*	Yes
UL	*Saccharomyces boulardii* CNCM I-745	1	*S. cerevisiae*	*S. cerevisiae*	Yes
V	*Bifidobacterium infantis* BI04 (*B. animalis* subsp. *lactis*)	7	*Bifidobacterium spp.*	*B. animalis*	Yes
	*Bifidobacterium longum* BL03 (*B. animalis* subsp. *lactis*)		*Bifidobacterium spp.*	*B. animalis*	Yes
	*Bifidobacterium breve* BB02		*Bifidobacterium spp.*	*Bifidobacterium* spp.	Yes
	*Lactobacillus acidophilus* BA05		*L. acidophilus*	*Lactobacillus spp.*	Yes
	*Lactobacillus delbrueckii* subsp. *bulgaricus* BD08 (*L. helveticus*)		*Lactobacillus spp.*	*L. delbrueckii*	Yes
	*Lactobacillus paracasei* BP07		*L. paracasei*	*Lactobacillus spp.*	Yes
	*Lactobacillus plantarum* BP06		*L. plantarum*	*L. plantarum*	Yes
	*Streptococcus thermophilus* BT01		*S.salivarius/thermophilus*	*S. salivarius*	Yes
Y	*Bifidobacterium animalis lactis*	8	*Bifidobacterium spp.*	*B. animalis*	Yes
	*Bifidobacterium brevis*		*Bifidobacterium spp.*	*Bifidobacterium spp.*	Yes
	*Enterococcus faecium*		*E. faecium*	*E. faecium*	Yes
	*Lactobacillus acidophilus*		*L. acidophilus*	*Lactobacillus spp.*	Yes
	*Lactobacillus delbrueckii* subsp. *bulgaricus*		*Lactobacillus spp.*	*L. delbrueckii*	Yes
	*Lactobacillus paracasei*		*-*	*Lactobacillus spp.*	Yes
	*Lactobacillus plantarum*		*L. plantarum*	*L. plantarum*	Yes
	*Streptococcus salivarius* subsp. *thermophilus*		*S. salivarius/thermophilus*	*S. salivarius*	Yes

Text in bold depicts strains of microorganisms claimed on the product label, but not identified in the study. AP, Align Probiotic Supplement Digestive Support; BF, Bioflorin; BG, Biotics G; CO, Colidis; CU, Culturelle Digestive Health Daily Probiotic; E4, Enterogermina 4B; E6, Enterogermina 6B; EP, Enterolactis Plus; ES, Enterogermina Sporattiva; FD, FlorMidabìl Daily; L, Linex; LP, Lactoflorene Plus; LR, Lactibiane Reference; MALDI-TOF MS, matrix-assisted laser desorption/ionization–time-of-flight mass spectrometry; NB, Nature’s Bounty Acidophilus Probiotic; O, Omnibiotic 10 AAD; PH, Phillips’ Daily Care Colon Health Daily Probiotic; R, RenewLife Ultimate Flora Women’s Care Probiotic; SD, Schiff Digestive Advantage; UL, Ultra-Levure 50; V, VSL#3; Y, Yovis.

### 3.2. Survival in ASTM- and USP-simulated gastric fluids

**Figure 2:**
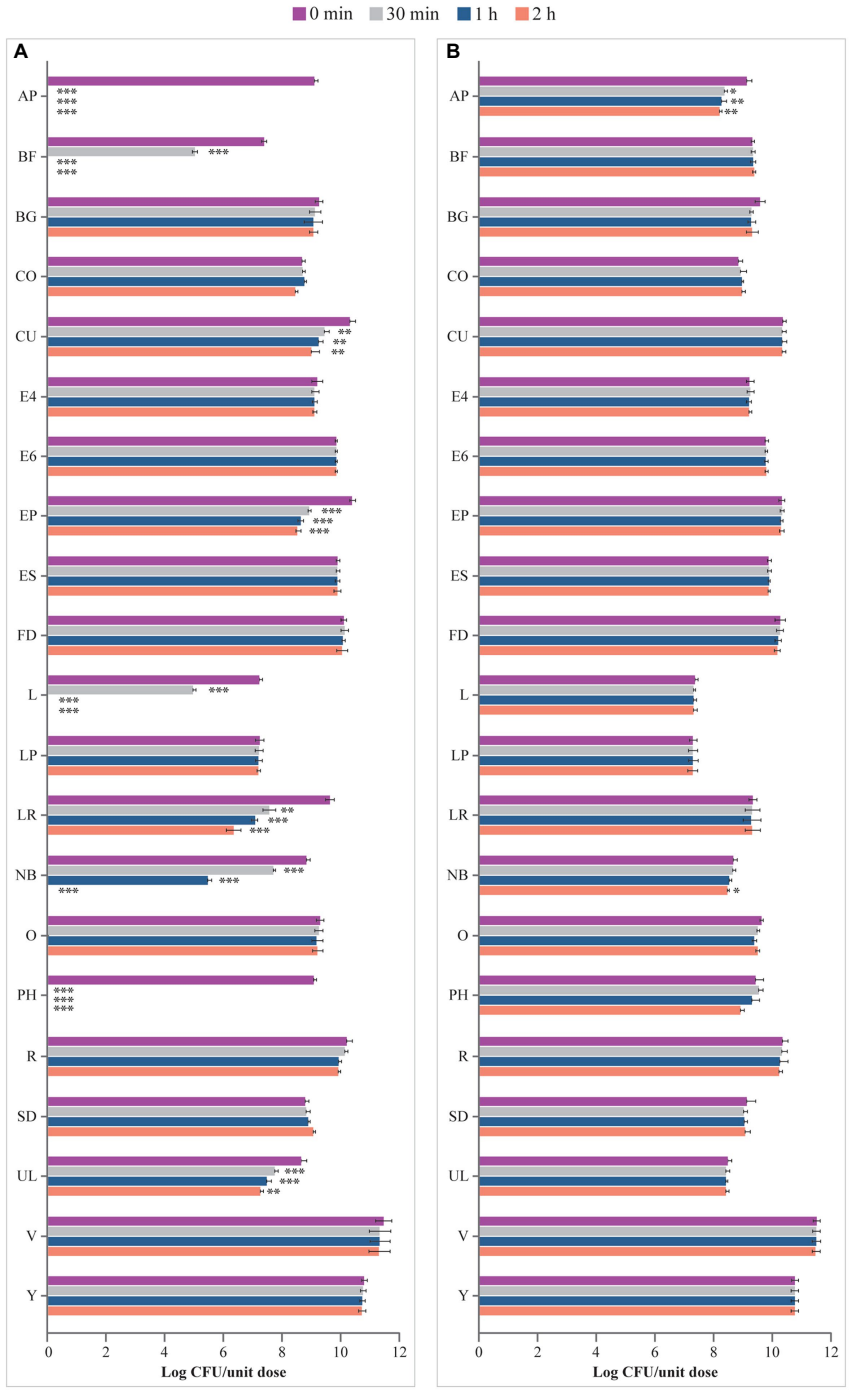
Viability (log CFU/unit dose) of microbes from probiotic products **(A)** in ASTM-simulated gastric fluid at pH 1.5 and **(B)** in ASTM-simulated gastric fluid at pH 3.0. AP, Align Probiotic Supplement Digestive Support; BF, Bioflorin; BG, Biotics G; CFU, colony-forming unit; CO, Colidis; CU, Culturelle Digestive Health Daily Probiotic; E4, Enterogermina 4B; E6, Enterogermina 6B; EP, Enterolactis Plus; ES, Enterogermina Sporattiva; FD, FlorMidabìl Daily; L, Linex; LP, Lactoflorene Plus; LR, Lactibiane Reference; NB, Nature’s Bounty Acidophilus Probiotic; O, Omnibiotic 10 AAD; PH, Phillips’ Daily Care Colon Health Daily Probiotic; R, RenewLife Ultimate Flora Women’s Care Probiotic; SD, Schiff Digestive Advantage; UL, Ultra-Levure 50; V, VSL#3; Y, Yovis Microbial counts were performed at 0 min, 30 min, 1 h, and 2 h of incubation and expressed as log CFU/unit dose. ***p < 0.001, **p < 0.01, and *p < 0.05 compared to 0 min.

As shown in Figure 2A, incubation in the ASTM-simulated gastric fluid at pH 1.5 for up to 2 h had no effect on microbes contained in BG, CO, E4, E6, ES, FD, LP, O, R, SD, V, and Y. In contrast, viability of the microorganisms contained in AP, BF, CU, EP, L, LR, NB, PH, and UL significantly decreased starting from 30 min of incubation in the fluid at pH 1.5. In addition, no residual living cells were obtained for AP, BF, L, NB, and PH at different times post inoculation compared to 0 min (AP, BF, EP, L, NB, PH: *p* < 0.001 at 30 min, 1 h, 2 h; CU: *p* < 0.01 at 30 min, 1 h, 2 h; LR: *p* < 0.01 at 30 min, *p* < 0.001 at 1 h and 2 h; UL: *p* < 0.001 at 30 min and 1 h, *p* < 0.01 at 2 h; Figure 2A).

Similarly, incubation in ASTM-simulated gastric fluid at pH 3.0 for up to 2 h showed no effect on the viability of microbes contained in most products (BF, BG, CO, CU, E4, E6, EP, ES, FD, L, LP, LR, O, PH, R, SD, UL, V, and Y; Figure 2B). However, a significant decline in the number of living microbes contained in AP after 30 min (*p* < 0.05 at 30 min, *p* < 0.01 at 1 h and 2 h) and NB after 2 h (*p* < 0.05) of incubation was observed.

**Figure 3:**
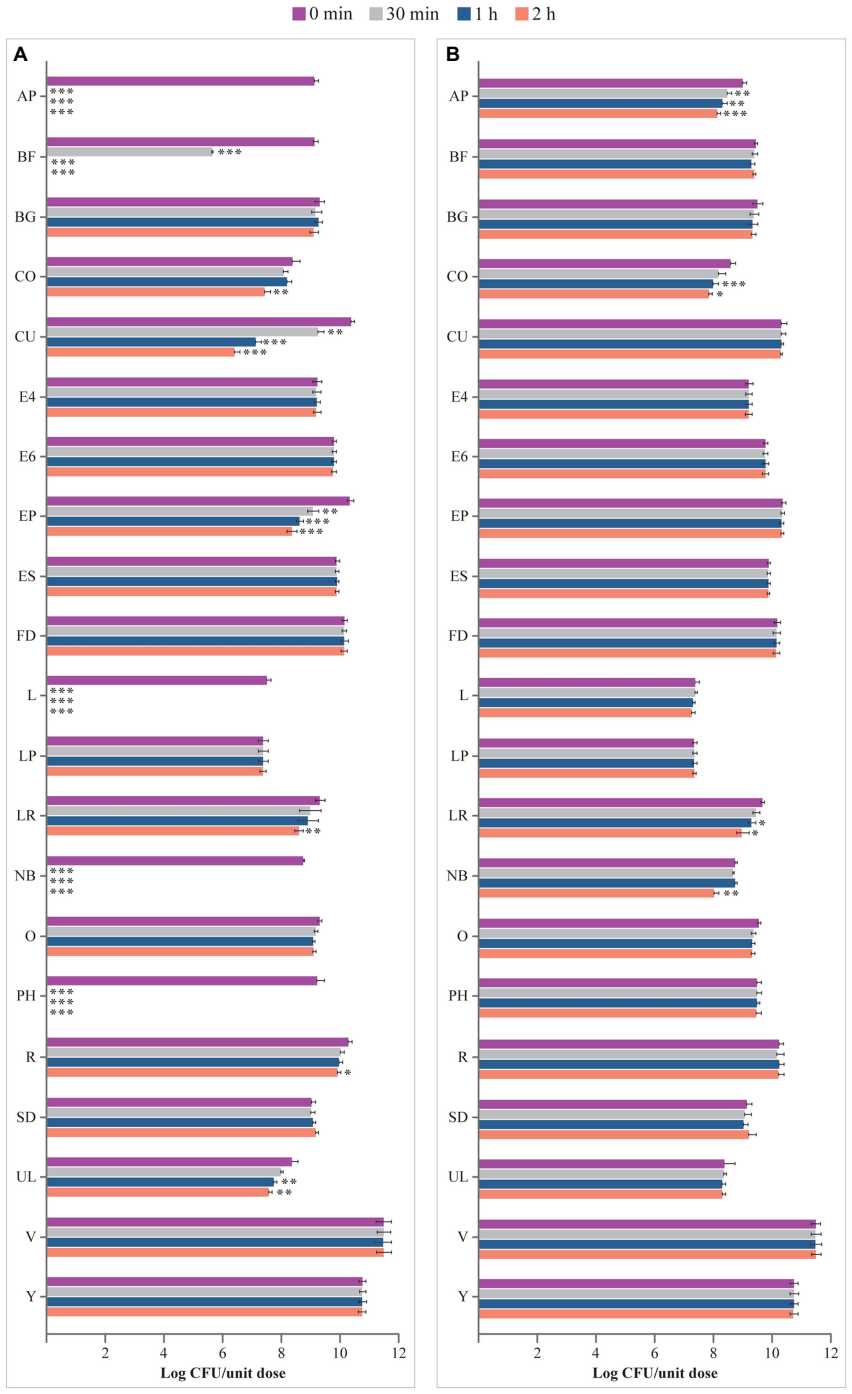
Viability (log CFU/unit dose) of microbes from probiotic products **(A)** in USP-simulated gastric fluid at pH 1.5 and **(B)** in USP-simulated gastric fluid at pH 3.0. AP, Align Probiotic Supplement Digestive Support; BF, Bioflorin; BG, Biotics G; CFU, colony-forming unit; CO, Colidis; CU, Culturelle Digestive Health Daily Probiotic; E4, Enterogermina 4B; E6, Enterogermina 6B; EP, Enterolactis Plus; ES, Enterogermina Sporattiva; FD, FlorMidabìl Daily; L, Linex; LP, Lactoflorene Plus; LR, Lactibiane Reference; NB, Nature’s Bounty Acidophilus Probiotic; O, Omnibiotic 10 AAD; PH, Phillips’ Daily Care Colon Health Daily Probiotic; R, RenewLife Ultimate Flora Women’s Care Probiotic; SD, Schiff Digestive Advantage; UL, Ultra-Levure 50; V, VSL#3; Y, Yovis Microbial counts were performed at 0 min, 30 min, 1 h, and 2 h of incubation and expressed as log CFU/unit dose. ***p < 0.001, **p < 0.01, and *p < 0.05 compared to 0 min.

Viability of microbes from products in USP-simulated gastric fluid at pH 1.5 is depicted in Figure 3A. Microbes contained in BG, E4, E6, ES, FD, LP, O, SD, V, and Y were not affected by the incubation in this fluid for up to 2 h compared with 0 min. Viability of the microorganisms of AP, BF, CO, CU, EP, L, LR, NB, PH, R, and UL significantly decreased at different times in the USP-simulated gastric juice at pH 1.5, with no residual living cells for AP, BF, L, NB, and PH at different times post incubation, compared with 0 min (AP, BF, L, NB, PH: *p* < 0.001 at 30 min, 1 h, and 2 h; CO, LR: *p* < 0.01 at 2 h; CU: *p* < 0.01 at 30 min, *p* < 0.001 at 1 h and 2 h; EP: *p* < 0.01 at 30 min, *p* < 0.001 at 1 h and 2 h; R: *p* < 0.05 at 2 h; UL: *p* < 0.01 at 1 h and 2 h).

USP-simulated gastric juice at pH 3.0 did not affect the viability of microbes contained in BF, BG, CU, E4, E6, EP, ES, FD, L, LP, O, PH, R, SD, UL, V, and Y, at 2 h versus at 0 min; see Figure 3B. In contrast, the number of microbes contained in AP, CO, LR, and NB significantly decreased at different times post inoculation compared to 0 min (AP: *p* < 0.01 at 30 min and 1 h, *p* < 0.001 at 2 h; CO: *p* < 0.001 at 1 h, *p* < 0.05 at 2 h; LR: *p* < 0.05 at 1 h and 2 h; NB: *p* < 0.01 at 2 h).

### 3.3. Survival in simulated intestinal fluids

**Figure 4:**
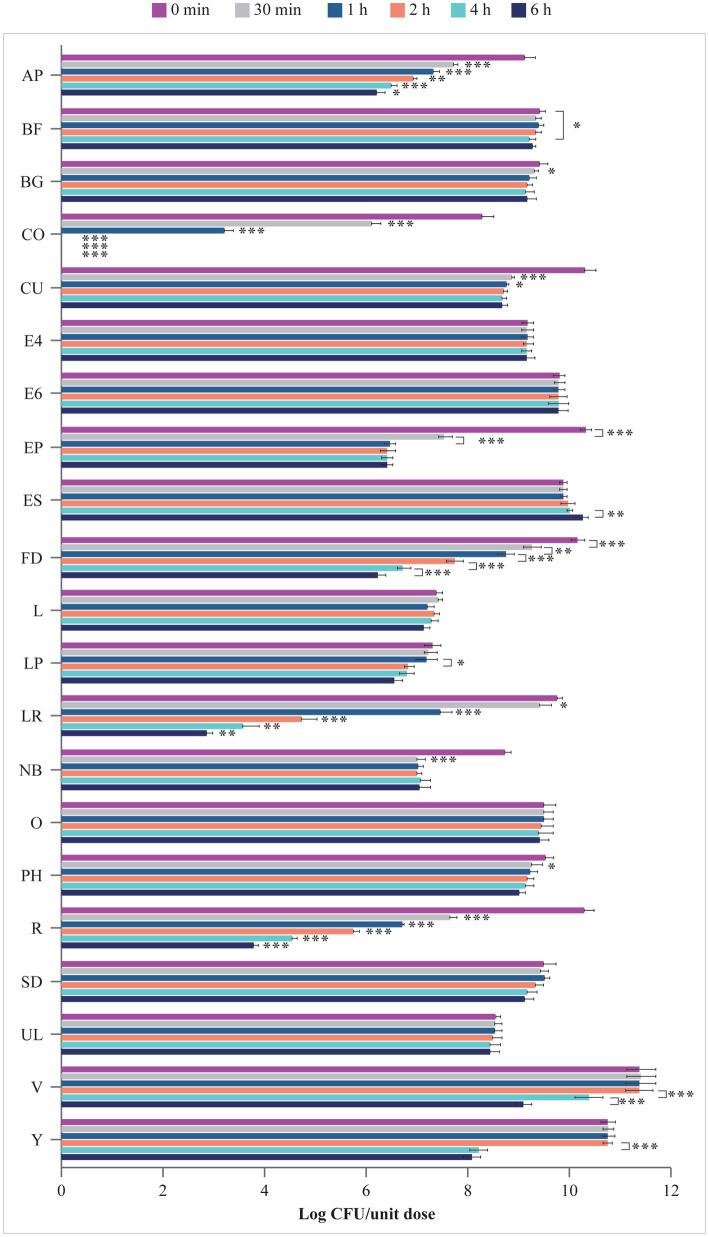
Viability (log CFU/unit dose) of microbes from probiotic products in simulated intestinal juice (pH 8.0). AP, Align Probiotic Supplement Digestive Support; BF, Bioflorin; BG, Biotics G; CFU, colony-forming unit; CO, Colidis; CU, Culturelle Digestive Health Daily Probiotic; E4, Enterogermina 4B; E6, Enterogermina 6B; EP, Enterolactis Plus; ES, Enterogermina Sporattiva; FD, FlorMidabìl Daily; L, Linex; LP, Lactoflorene Plus; LR, Lactibiane Reference; NB, Nature’s Bounty Acidophilus Probiotic; O, Omnibiotic 10 AAD; PH, Phillips’ Daily Care Colon Health Daily Probiotic; R, RenewLife Ultimate Flora Women’s Care Probiotic; SD, Schiff Digestive Advantage; UL, Ultra-Levure 50; V, VSL#3; Y, Yovis Microbial counts were performed at 0 min, 30 min, 1 h, 2 h, 4 h, and 6 h of incubation and expressed as log CFU/unit dose. Only significant *p* values highlighting the behavior of each product over time are shown: *p < 0.05, **p < 0.01, and ***p < 0.001.

Viability of microbes in the simulated intestinal fluid is shown in Figure 4. Microbes contained in E4, E6, L, O, SD, and UL survived in the artificial intestinal juice at pH 8.0 for up to 6 h. In contrast, the number of microorganisms of AP, BF, BG, CU, EP, FD, LP, LR, NB, PH, R, V, and Y significantly declined at different times post inoculation. The viability of microbes contained in CO progressively decreased starting from 30 min (*p* < 0.001 at 30 min vs. 0 min and 1 h vs. 30 min) with no residual living cells starting from 2 h (*p* < 0.001 vs. 1 h). Interestingly, while no variation in the log CFU/unit dose of ES was recorded up to 4 h, a significant increase in the number of microbes contained in this product was observed at 6 h (*p* < 0.01 vs. 4 h).

### 3.4. Summary of properties of the analyzed probiotic formulations

A summary of properties of the analyzed formulations with respect to compliance with the label claims and resistance to simulated gastrointestinal conditions is presented in Table 4.

**Table 4:** Summary of the properties of the analyzed probiotic products: compliance with the label claims for the number of living microbes and microbial composition, as well as resistance to simulated gastric and intestinal conditions.

			Resistance in				
Product	Compliance (CFU/unit dose)	Compliance (microbial composition)	ASTM pH 1.5	ASTM pH 3.0	USP pH 1.5	USP pH 3.0	Intestinal juice (pH 8.0)
AP	+	+	−	−	−	−	−
BF	+	+	−	+	−	+	−
BG	+	+	+	+	+	+	−
CO	+	+	+	+	−	−	−
CU	+	+	−	+	−	+	−
E4	+	+	+	+	+	+	+
E6	+	+	+	+	+	+	+
EP	+	+	−	+	−	+	−
ES	+	+	+	+	+	+	+*
FD	+	+	+	+	+	+	−
L	+	+	−	+	−	+	+
LP	−	+	+	+	+	+	−
LR	+	−	−	+	−	−	−
NB	+	+	−	−	−	−	−
O	+	−	+	+	+	+	+
PH	+	+	−	+	−	+	−
R	+	+	+	+	−	+	−
SD	+	+	+	+	+	+	+
UL	ND^#^	+	−	+	−	+	+
V	+	+	+	+	+	+	−
Y	+	+	+	+	+	+	−

* The product also showed growth of microorganisms; # not declared.

AP, Align Probiotic Supplement Digestive Support; ASTM, American Society for Testing and Materials; BF, Bioflorin; BG, Biotics G; CO, Colidis; CU, Culturelle Digestive Health Daily Probiotic; E4, Enterogermina 4B; E6, Enterogermina 6B; EP, Enterolactis Plus; ES, Enterogermina Sporattiva; FD, FlorMidabìl Daily; L, Linex; LP, Lactoflorene Plus; LR, Lactibiane Reference; NB, Nature’s Bounty Acidophilus Probiotic; O, Omnibiotic 10 AAD; PH, Phillips’ Daily Care Colon Health Daily Probiotic; pH, potential of hydrogen; R, RenewLife Ultimate Flora Women’s Care Probiotic; SD, Schiff Digestive Advantage; UL, Ultra-Levure 50; USP, United States Pharmacopeia; V, VSL#3; Y, Yovis.

## 4. Discussion

The probiotic market is growing worldwide, and products are continuously being monitored for their quality compared with their label claims. As the probiotics pass through varied environments starting from the acidic pH of the stomach to the alkaline pH of the intestine, where they are known to provide their benefits, evaluating their survival in the acidic and alkaline pH is also essential.

Leading probiotic brands commercialized in various countries as of 2019 were selected for this *in vitro* study. The specific products were prioritized based on health claims relevant to digestive health and considering their availability for purchase online. The study results demonstrated that most of the probiotics were compliant with their label claims, in terms of number and species of contained microorganisms. Probiotic LP analyzed in this study contained a lower number of viable microbes than that described on the product label. Earlier studies have highlighted that some commercially available probiotics did not contain the declared amount of living microorganisms (Lin et al., 2006; Kolaček et al., 2017; Vecchione et al., 2018). This may be attributed to conditions of manufacturing, packaging, and handling that can impact the viability of probiotic microbes (Fenster et al., 2019), thus potentially determining an overall reduction in beneficial effects (Kolaček et al., 2017). In fact, a reduced number of viable cells can be due to industrial processes that can stress some cells in becoming viable but not culturable. However, the quantification of microbes contained in the multispecies product LP can also be negatively affected by the cultural conditions adopted in this study. In contrast, probiotic BF contained a higher number of microbes in comparison with the labeled dose. It was considered acceptable as overage amounts of microbes are commonly included by manufacturers in probiotic supplements to ensure the presence of the labeled dose until the expiration date (Fenster et al., 2019).

In this study, product LR was not compliant with the label with respect to species *B. longum* and *L. helveticus* and product O did not contain the species *B. longum*. Earlier studies have also shown a disparity in results for the composition of some probiotic brands compared with the product label (Fasoli et al., 2003; Zawistowska-Rojek et al., 2016; Vecchione et al., 2018; Korona-Glowniak et al., 2019). One of the reasons for this inconsistency could be the lack of use of up-to-date methodologies for identification and quantification of microbes in probiotic formulations. In fact, the only use of culture-dependent methods is successful if all microbes contained in probiotic products can grow *in vitro*, which may be a challenge in multispecies formulations (Mazzantini et al., 2021). On the other hand, culture-independent methods can identify microbial species contained in the products (Marcobal et al., 2008), but are unable to establish their viability (Mazzantini et al., 2021). Because of the limitations in both types of methods, a combination of these may help in accurate identification of microbes in probiotic formulations. One of the highlights of the present study is the use of a combination of culture-dependent and culture-independent methods for the identification of microbes as reported in a few studies (Vecchione et al., 2018; Ullah et al., 2019).

In this study, contents of all the probiotic products were suspended in simulated GI fluids, irrespective of the dosage form, resulting in a wide variability in the microbial survival. To improve survivability and stability of the bacterial strains that are susceptible to a highly acidic or alkaline environment, some probiotic products are often produced in an encapsulated form.

Among microorganisms contained in encapsulated products, microbes of AP and BF did not withstand the simulated harsh gastric and intestinal environments once removed from capsules, leading to loss of viability.

Of the 21 probiotics analyzed in this study, four products, i.e., E4, E6, ES, and SD, were concordant with their product labels and survived in both gastric and intestinal environments. All the four products contained spores of *Bacillus* species. This finding is in line with previous studies reporting that spores of the genus *Bacillus*, especially *Bacillus clausii* (Ghelardi et al., 2015), are extremely resistant to the harsh GI conditions and can therefore successfully exert their beneficial effects in the GI tract (Cutting, 2011; Ghelardi et al., 2015; Elshaghabee et al., 2017; Lee et al., 2019).

An interesting observation in this study was that spores of *Bacillus clausii* contained in the product ES appeared to germinate and grow vegetative cells in the artificial alkaline environment. An earlier study has also demonstrated that *Bacillus clausii* spore combination of O/C, N/R, SIN, T strains can germinate and actively multiply in the intestinal fluid beyond the initial dose, possibly because of the alkaliphilic nature of *Bacillus* species (Vecchione et al., 2018). However, in this study, products E4 and E6 that contain *Bacillus clausii* spores appeared incapable of growing in the studied conditions despite showing similar stability in intestinal fluid. One possible reason is the difference in formulations: E4 and E6 have higher initial spore content in vials and lyophilized powder, respectively, than previously tested formulations only containing 2 billion CFUs in vials. Moreover, ES contains only single-strain *Bacillus clausii* in orodispersible granules. These differences may have affected the dynamics of the strains overall in the setup.

Of note, product LP also contained a *Bacillus* species, *B. coagulans*. Nevertheless, overall viability count of microorganisms in the product was found to be reduced in the simulated intestinal juice. One of the possible reasons may be that other species included in this multispecies formulation (*Bifidobacterium animalis* subspecies *lactis*, *Lactobacillus acidophilus*, and *Lactobacillus casei*) are susceptible to simulated intestinal conditions, thus affecting the overall viability count.

The merits of this *in vitro* study as compared with clinical trials are inherent to any other *in vitro* study, i.e., control over independent variables and unforeseen bias and ease of operation, thus improving internal validity of the results. A wide range of probiotics commercialized worldwide were included in this study as compared with previous studies performed on products majorly available in Italy or those marketed in specific countries and containing specific bacterial strains (Masco et al., 2005; Vecchione et al., 2018; Korona-Glowniak et al., 2019). Further, a combination of culture-dependent and -independent methods used for the identification and quantification of microbes aided to yield more accurate results.

On the other hand, the use of artificial fluids in this study did not consider the influence of dietary and other non-acid constituents of gastric secretions. However, it had the benefit of not being restricted by the availability of animal-derived material and provided more controllable and homogenous experimental conditions to compare the effect of an acidic or alkaline environment on different probiotic products.

## 5. Conclusion

This *in vitro* study demonstrated that 18 out of the 21 probiotics available worldwide were compliant with the number and species of the microbes described on their product labels, thus indicating a general high quality of commercialized probiotic products in terms of microbial composition. The viability of microbes in simulated gastric and intestinal environments showed variability because of peculiar properties of each microbial strain and settings of probiotic formulations themselves. *Bacillus clausii* tend to perform well in these survivability tests because of their spore-forming capabilities. Although the results obtained in this study indicate a good quality of the tested formulations, it is important to stress that stringent quality controls of probiotic products should always be performed to provide optimal health benefits for the host.

## Data availability statement

The original contributions presented in the study are included in the article/Supplementary Material. Further inquiries can be directed to the corresponding author.

## Author contributions

EG and MP contributed to the concept and design, manuscript revision, and approval of submitted version. DM, FC, MC, and AP contributed to the methodologies, data analyses, manuscript revision, and approval of submitted version. AL contributed to the revision of data and results, manuscript revision, and approval of submitted version. BF contributed to the statistical planning, manuscript revision, and approval of submitted version. All authors take full accountability for the work and all the content and editorial decisions.

## Funding

The authors declare that this study received funding from Sanofi. The funder was involved in study design, preparation of the manuscript and decision to publish.

## Conflict of interest

MP and BB are employees of Sanofi and may hold shares and/or stock options in the company. EG has been a lecturer for Sanofi.

The remaining authors declare that the research was conducted in the absence of any commercial or financial relationships that could be construed as a potential conflict of interest.

## Publisher’s note

All claims expressed in this article are solely those of the authors and do not necessarily represent those of their affiliated organizations, or those of the publisher, the editors and the reviewers. Any product that may be evaluated in this article, or claim that may be made by its manufacturer, is not guaranteed or endorsed by the publisher.

## Footnotes

^1^ https://www.arb-silva.de
