# Supplementary material for: Analysis of the microbial content of probiotic products commercialized worldwide and survivability in conditions mimicking the human gut environment
Source: Front Microbiol. 2023 May 5;14:1127321. doi: 10.3389/fmicb.2023.1127321 (PMC10208119; doi:10.3389/fmicb.2023.1127321)
Supplement: Supplementary file 1 [file Data_Sheet_1.docx]

**Supplementary Material**

**Supplementary Table 1.** Primers used for amplification of the 16S and 18S coding regions

| Region | Amplicon length  (bp) | Primer name and sequence (5′-3′) |
| --- | --- | --- |
| Bacterial 16S V3–V4 466 | 466 | 341F - CCTAYGGGRBGCASCAG  806R - GGACTACNNGGGTATCTAAT |
| Eukaryotic 18S V4 350 | 350 | 528F - GCGGGTAATTCCAGCTCCAA  706R - AATCCRAGAATTTCACCTCT |

**Supplementary Figure 1.** A sequential approach for testing hypotheses to determine the resistance to artificial gastric juice and to handle the multiple comparisons


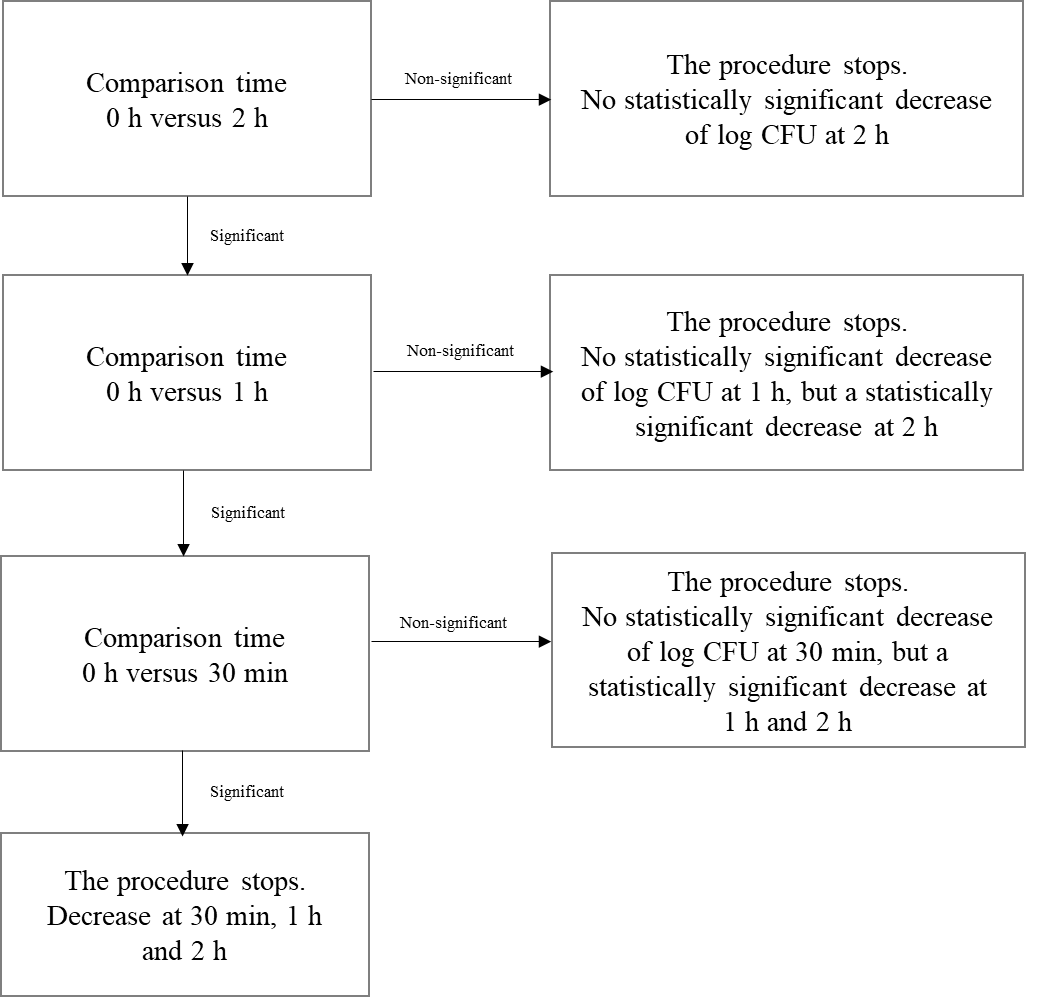


CFU, colony-forming unit; h, hour; min, minute
